# Retinoic acid inducible gene I Activates innate antiviral response against human parainfluenza virus type 3

**DOI:** 10.1186/1743-422X-6-200

**Published:** 2009-11-17

**Authors:** Ahmed Sabbah, Santanu Bose

**Affiliations:** 1 Department of Microbiology and Immunology, The University of Texas Health Science Center at San Antonio, San Antonio, Texas, USA

## Abstract

Human parainfluenza virus type 3 (HPIV3) is a respiratory paramyxovirus that infects lung epithelial cells to cause high morbidity among infants and children. To date, no effective vaccine or antiviral therapy exists for HPIV3 and therefore, it is important to study innate immune antiviral response induced by this virus in infected cells. Type-I interferons (IFN, interferon-α/β) and tumor necrosis factor-α (TNFα activated by NFκB) are potent antiviral cytokines that play an important role during innate immune antiviral response. A wide-spectrum of viruses utilizes pattern recognition receptors (PRRs) like toll-like receptors (TLRs) and RLH (RIG like helicases) receptors such as RIGI (retinoic acid inducible gene -I) and Mda5 to induce innate antiviral response. Previously it was shown that both TNFα and IFNβ are produced from HPIV3 infected cells. However, the mechanism by which infected cells activated innate response following HPIV3 infection was not known. In the current study, we demonstrated that RIGI serves as a PRR in HPIV3 infected cells to induce innate antiviral response by expressing IFNβ (via activation of interferon regulatory factor-3 or IRF3) and TNFα (via activation of NF-κB).

## Findings

Human parainfluenza virus type 3 (HPIV3) is an enveloped non-segmented negative-sense single stranded (NNS) RNA virus that belongs to the paramyxovirus family [1]. HPIV3 is a lung tropic virus known to cause severe respiratory diseases (croup, bronchiolitis, pneumonia) in infants, children, elderly and immuno-comprimised individuals [1,2]. Although HPIV3 causes life-threatening respiratory tract diseases, currently no effective vaccine or antiviral therapy exists. Therefore, elucidation of innate immune antiviral response induced by HPIV3 holds significant potential for development of effective antiviral therapies in the near future. The innate immune antiviral response against viruses represents an important host defense mechanism [3]. Innate immunity comprises the first line of defense by the host to combat virus infection before an orchestrated adaptive immune response is launched. Two key molecules regulating the innate antiviral function are interferon regulatory factors (IRFs) and NFκB [3]. These two transcription factors are activated either individually or together in infected cells, resulting in the expression and production of potent antiviral cytokines IFN-α/β (IFN) (type I interferon) [4,5]. IFNα/β produced from infected cells binds to their cognate IFN receptors on uninfected cells to induce the JAK/STAT antiviral pathway. Thus the paracrine action of IFN is absolutely critical during innate antiviral defense [4-8]. Although the paracrine action of IFNs plays a critical role in innate immune antiviral response, we have also identified an IFN independent antiviral pathway against HPIV3 and human respiratory syncytial virus (RSV) that was dependent on NF-κB activation and production of pro-inflammatory cytokines like tumor necrosis factor-α (TNFα) [9,10].

Infected cells utilize pattern recognition receptors (PRRs) to recognize pathogen (virus) associated molecular patterns (PAMPs) to trigger activation of the transcription factors IRF3 and NF-κB, which then translocate to the nucleus to transactivate antiviral genes like IFNα/β, TNFα etc [11]. So far two classes of viral PRRs have been identified - toll-like receptors (TLRs) [12] and RLH (RIG like helicases) receptors such as RIGI (retinoic acid inducible gene - I) and Mda5 [13]. Recently, we also demonstrated that NOD-like receptors such as NOD2 could act as a PRR for RSV and influenza A virus [14]. TLRs are type I integral transmembrane proteins that are utilized by various viruses to activate NFκB and IRF3 in infected cells. Majority of TLRs require MyD88 as an adaptor protein to induce TLR-dependent signaling [12]. In contrast to membrane bound TLR proteins, RLH receptors are cytoplasmic PRRs. Both RIGI and Mda5 signals antiviral response via induction of IRF3 and NFκB pathways after binding to the single stranded RNA genome of paramyxo, orthomyxo and picarnoviruses [13]. Although TLRs and RLH receptors were shown to induce innate antiviral response following infection with various RNA and DNA viruses, the mechanism by which HPIV3 activates the innate response is not known. Moreover, there are no reports of any PRR that play an important role in activation of IRF3/NF-κB during HPIV3 infection.

The innate immune response to HPIV3 is not well understood. HPIV3 has been shown to produce IFN-I *in vivo *and *in vitro *[15-17] yet no reports of IRF3 activation by HPIV3 exist. In addition, previous studies have demonstrated that HPIV3 activates NFκB to produce TNFα for establishment of antiviral state [9]. Moreover, TLRs may be involved in NFκB activation (at 24 h post-infection) since blocking MyD88 function diminished NF-κB activation in HPIV3 infected cells by 50%-55%. This suggested that both TLRs and non-TLR molecules may be involved in NFκB activation in HPIV3 infected cells. A similar scenario has been reported for another paramyxovirus, human respiratory syncitial virus (RSV). It was shown that both TLR3 and RIGI are involved in NF-κB activation following RSV infection [18]. Therefore, we investigated whether similar to RSV; HPIV3 can also utilize RIGI to activate innate antiviral response. In the current studies we have demonstrated that in A549 (A549 cells are human respiratory epithelial cells that have been routinely used as a model of type II alveolar epithelial cells) cells RIGI plays an important role in activation of innate antiviral response during HPIV3 infection. Moreover, RIGI was involved in activating both arms (NFκB/TNFα and IRF3/IFNα/β) of innate immunity following HPIV3 infection.

**Figure 1:**
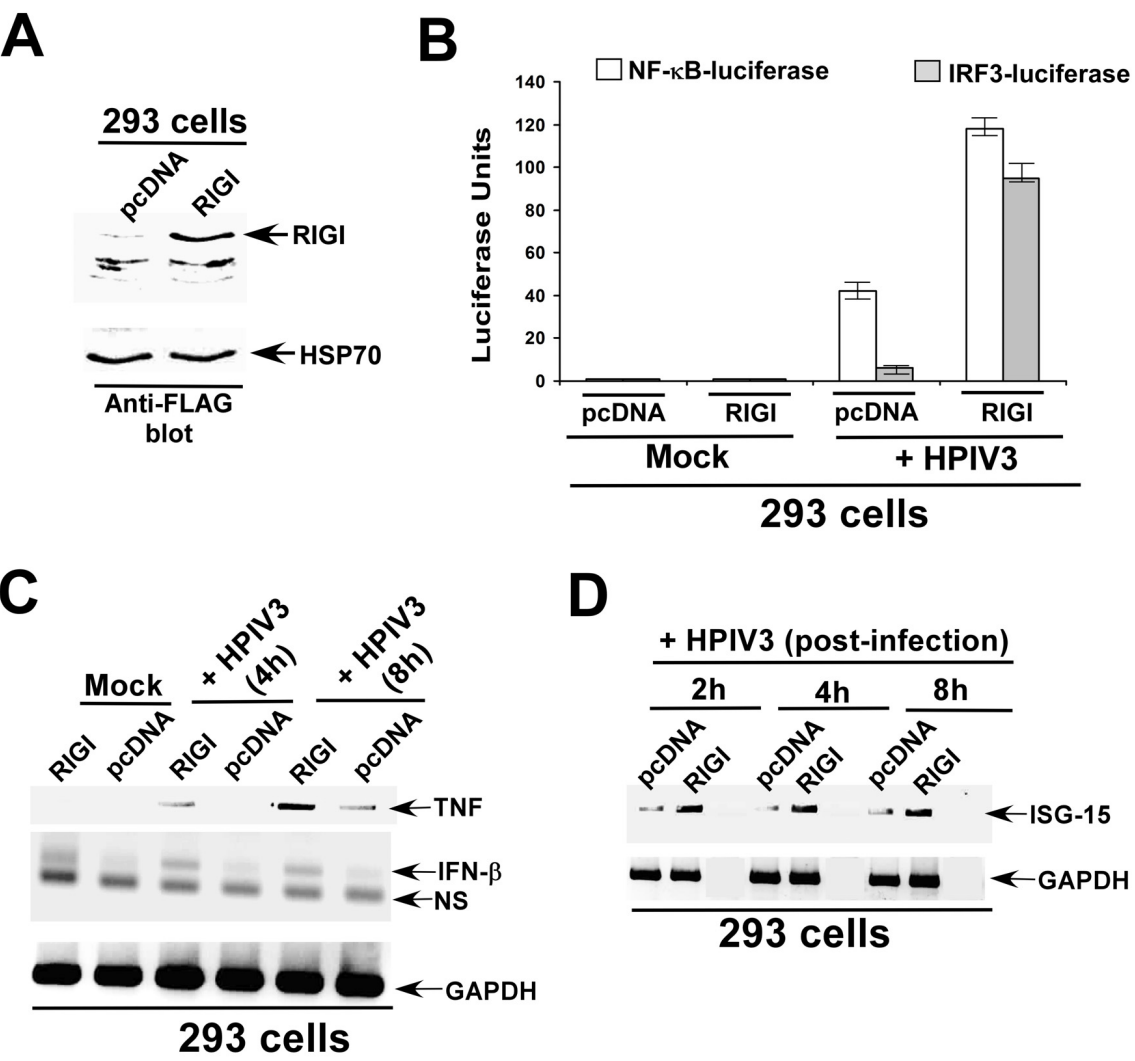
**Activation of antiviral response by RIGI during HPIV3 infection**. **(a) **Western blot analysis (with anti-FLAG antibody) of RIGI expression in 293 cells transfected with pcDNA and RIGI (FLAG tagged) plasmids. **(b) **Activation of IRF3-luciferase and NF-κB-luciferase in mock and HPIV3 infected (at 8 h post-infection) 293 cells transfected with either pcDNA or RIGI. The luciferase assay results are presented as mean ± standard deviation from three independent experiments. **(c) **RT-PCR analysis of TNF and IFN-β expression (at 4 h and 8 h post-infection) in mock and HPIV3 infected 293 cells transfected with either pcDNA or RIGI. GAPDH served as a loading control. NS; non-specific. **(d) **RT-PCR analysis of ISG-15 expression (at 2 h, 4 h and 8 h post-infection) in mock and HPIV3 infected 293 cells transfected with either pcDNA or RIGI

In order to study the involvement of RIGI in antiviral response stimulation via activation of IRF3/NFκB, we expressed RIGI (FLAG tagged) in 293 cells (these cells does not express majority of endogenous PRRs) and analyzed IFN/NFκB activation following HPIV3 infection. For these experiments, 293 cells were transfected (by using lipofectamine 2000 from Invitrogen) with FLAG-RIGI, pcDNA, NF-κB-luciferase, and IRF3-luciferase plasmids. At 24 h post-trasfection, cells were infected with HPIV-3 (0.5 MOI) and luciferase assay was performed at 8 h post-infection as described previously [9,14]. The efficiency of RIGI expression was confirmed by Western blotting (with FLAG antibody) of lysate obtained from RIGI-FLAG transfected 293 cells (Fig. 1A). As shown, in Fig. 1B, RIGI expression resulted in drastic activation (measured by luciferase assay) of both NF-κB and IRF3 in HPIV3 infected cells. The role of RIGI in activation of these transcription factors were further confirmed by detecting expression [reverse transcription or RT-PCR was performed using RedMIX Plus (Gene Choice) with the following primers: *GAPDH forward, 5'*-GTCAGTGGTGGACCTGACCT, *GAPDH reverse, 5'*-AGGGGTCTACATGGCAACTG; *ISG15 forward, 5'*-CCGTGAAGATGCTGGCG, *ISG15 reverse, 5'*-CGAAGGTCAGCCAGAAC; *IFN-β forward, 5'*-GATTCATCTAGCACTGGCTGG, *IFN-β reverse*, 5'CTTCAGGTAATGCAGAATCC; *TNF-α forward*, GAGTGACAAGCCTGTAGCCCATGTTGTAGCA, *TNF-α reverse*, GCAATGATCCCAAAGTAGACCTGCCCAGACT] of their target genes, TNF, IFN-β and ISG-15 (interferon stimulated gene-15) in infected 293 cells expressing RIGI (Fig. 1C, 1D). These results demonstrated that RIGI is capable of activating an innate antiviral response in HPIV3 infected cells.

**Figure 2:**
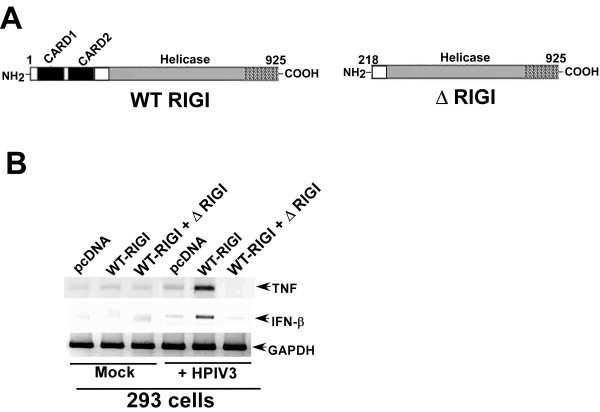
**CARD domains of RIGI are important for activation of antiviral response**. **(a) **A schematic showing the RIGI constructs; WT RIGI (wild type RIGI)), ΔRIGI (RIGI lacking the two CARD domains). **(b) **RT-PCR analysis of TNF and IFN-β expression (at 8 h post-infection) in mock and HPIV3 infected 293 cells transfected with the indicated plasmids. GAPDH served as a loading control.

RIGI protein consists of helicase and two CARD (caspase recruitment domain) domains (Fig. 2A) domains. Previous studies have shown that CARD domains are required for RIGI mediated signal transduction [19]; which constitutes interaction of RIGI with the mitochondrial localized adaptor protein MAVS (IPS-1) and activation of IRF3 and NF-κB [18]. In order to investigate the role of caspase domains in antiviral signaling, we co-expressed FLAG tagged wild type (WT) and CARD deleted RIGI (ΔRIGI) in 293 cells, followed by HPIV3 infection. At 8 h post-infection, TNF and IFN-β induction was measured by RT-PCR. Our result revealed that CARD domains are critical for antiviral signaling, since co-expression of WT and ΔRIGI resulted in loss of IFN-β and TNF induction in infected cells (Fig. 2B). These results demonstrated that CARD domains are important for RIGI signaling during HPIV3 infection. Furthermore, RIGI lacking the CARD domains (i.e. ΔRIGI) can act as a dominant negative molecule to suppress the activity of functional RIGI during HPIV3 infection.

**Figure 3:**
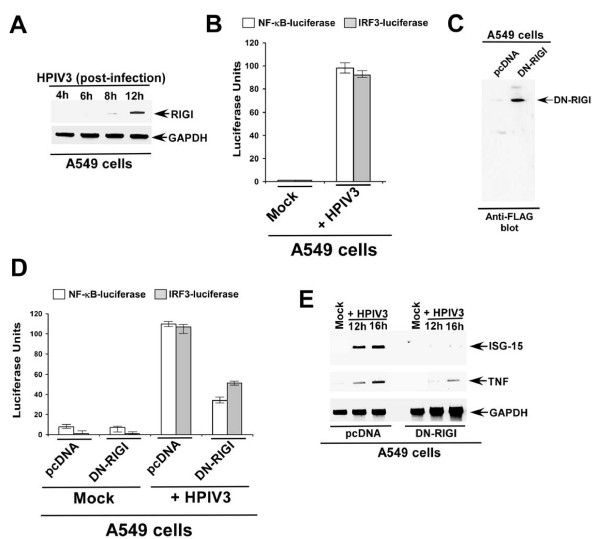
**Inactivation of endogenous RIGI in A549 cells by dominant-negative RIGI (ΔRIGI) abrogates innate antiviral response**. **(a) **RT-PCR analysis of endogenous RIGI expression in HPIV3 infected (at 4 h-12 post-infection) A549 cells. **(b) **Activation of IRF3-luciferase and NF-κB-luciferase in mock and HPIV3 infected (at 12 h post-infection) A549 cells. The luciferase assay results are presented as mean ± standard deviation from three independent experiments. **(c) **Expression of FLAG tagged dominant-negative RIGI (DN-RIGI) in A549 cells was assessed by Western blotting A549 cell (transfected with either pcDNA or FLAG-DN-RIGI) lysate with anti-FLAG antibody. **(d) **Activation of IRF3-luciferase and NFκ-B-luciferase in mock and HPIV3 infected (at 12 h post-infection) A549 cells trasfected with either pcDNA or DN-RIGI. The luciferase assay results are presented as mean ± standard deviation from three independent experiments. **(e) **RT-PCR analysis of TNF and ISG-15 expression (at 12 h and 16 h post-infection) in mock and HPIV3 infected A549 cells transfected with either pcDNA or DN-RIGI.

Since HPIV3 is a respiratory virus, we next evaluated the role of RIGI in inducing IRF3/NF-κB in infected human lung epithelial A549 cells. Infection of A549 cells with HPIV3 resulted in induction of endogenous RIGI expression (RT-PCR was performed using the following primers: *RIGI forward, 5'*-GCATATTGACTGGACGTGGCA, *RIGI reverse, 5'*-CAGTCATGGCTGCAGTTCTGTC) during relatively early infection time frame (8 h-12 h post-infection) (Fig. 3A). Similarly, IRF3 and NF-κB (as assessed by luciferase assay of infected A549 cells transfected with IRF3 and NF-κB luciferase) was induced by HPIV3 during early infection (within 12 h post-infection) (Fig. 3B). Based on similar induction/activation kinetics of RIGI and IRF3/NF-κB, we speculated that endogenous RIGI may play a role during IRF3/NF-κB activation. To examine such role of RIGI, we initially utilized A549 cells expressing (following transfection) ΔRIGI-FLAG, since ΔRIGI acted as a dominant-negative molecule (Fig. 2). Efficient expression of ΔRIGI (denoted as dominant negative RIGI or DN-RIGI) is evident from the Western blotting of A549 cell lysate with FLAG antibody (to detect ΔRIGI-FLAG) (Fig. 3C). In order to investigate the involvement of RIGI, A549 cells were transfected with ΔRIGI-FLAG, NF-κB- or IRF3-luciferase. At 24 h post-transfection, cells were infected with HPIV3 and luciferase activity was measured at 12 h post-infection. Expression of ΔRIGI in A549 resulted in drastic decline in both NF-κB and IRF3 activity following HPIV3 infection (Fig. 3D). In addition, infected cells expressing ΔRIGI lost expression of IRF3/NF-κB target genes TNF and ISG-15 (Fig. 3E). These results demonstrated that endogenously expressed RIGI plays a crucial role in activation of innate antiviral response following HPIV3 infection of human lung epithelial cells.

**Figure 4:**
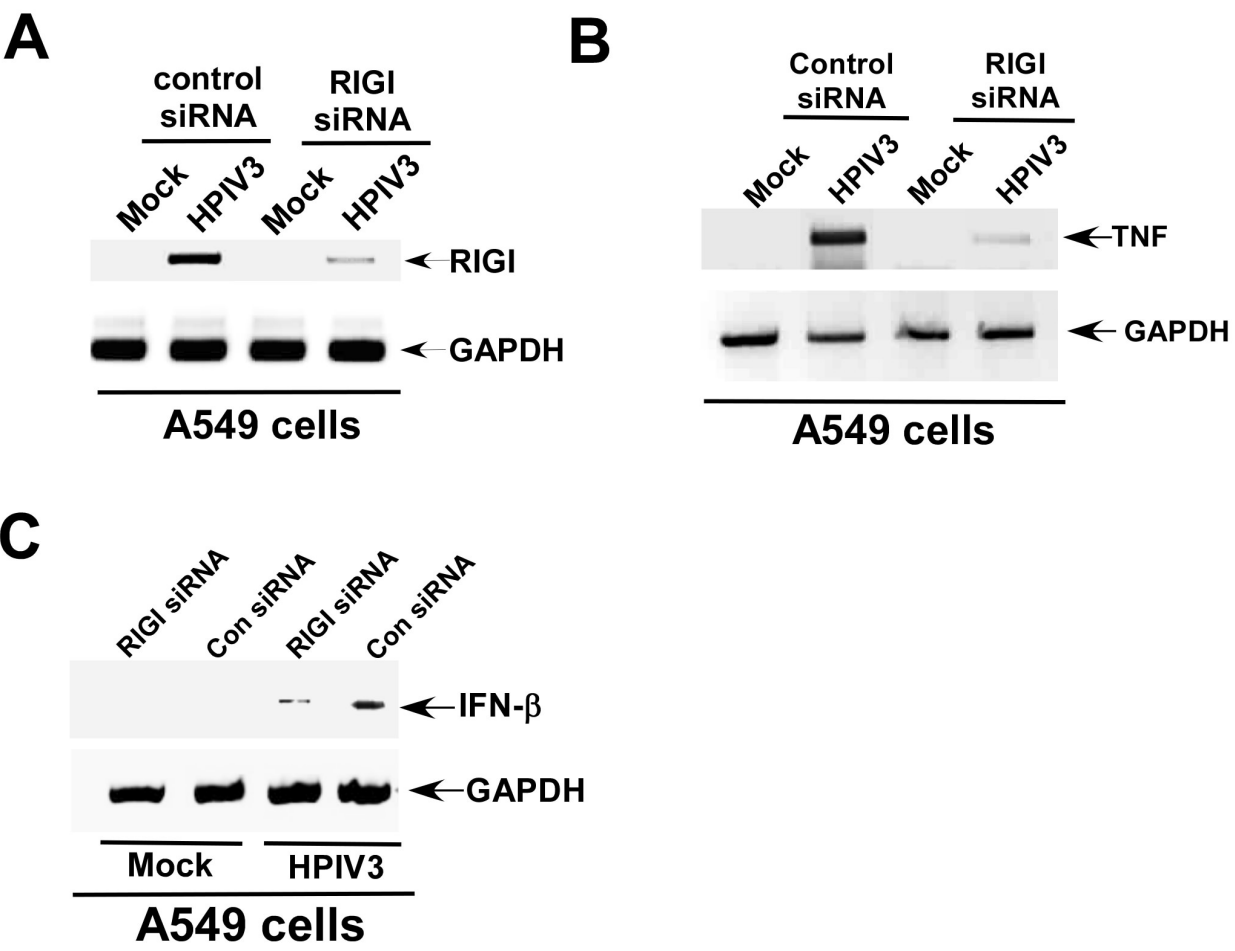
**Silencing of endogenous RIGI in A549 cells results in loss of induction of antiviral response**. **(a) **RT-PCR analysis of RIGI expression in mock and HPIV3 infected (at 12 h post-infection) A549 cells transfected with either control siRNA or RIGI specific siRNA. **(b) **RT-PCR analysis of TNF expression in mock and HPIV3 infected (at 12 h post-infection) A549 cells transfected with either control siRNA or RIGI specific siRNA. **(c) **RT-PCR analysis of IFN-β expression in mock and HPIV3 infected (at 12 h post-infection) A549 cells transfected with either control siRNA (Con siRNA) or RIGI specific siRNA (RIGI siRNA).

The role of endogenous RIGI was further confirmed by silencing RIGI expression in A549 cells. The silencing was performed by transfecting A549 cells with control or RIGI specific siRNA [negative control siRNA and DDX58-1 (RIG-I) siRNA were ordered from Qiagen and cells were transfected with 80 nM of siRNA using lipofectamine 2000]. The efficiency of silencing was validated in A549 cells, since HPIV3 failed to induce RIGI expression in cells expressing RIGI-specific siRNA (Fig. 4A). We next utilized the RIGI silenced A549 cells to examine the role of RIGI. Analysis of TNF gene expression by RT-PCR revealed failure of HPIV3 to optimally induce TNF in RIGI silenced cells, compared to cells transfected with control siRNA (Fig. 4B). Similarly, expression of IFN-β following HPIV-3 infection was drastically reduced in the absence of RIGI (RIGI silenced cells) protein (Fig. 4C). These results demonstrated an involvement of endogenous RIGI in inducing the innate antiviral pathway in HPIV3 infected human lung cells.

Our results have demonstrated a role of RIGI as a HPIV3 specific PRR involved in inducing innate antiviral response following activation of IRF3/IFN and NF-κB/TNF. However, additional non-RIGI PRRs may also be involved in inducing innate response following HPIV3 infection. In that context, well-established PRRs like Mda5, TLR3 and TLR7 that are known to recognize viruses and single-stranded RNA (ssRNA) are not involved during innate response by HPIV3 infected lung epithelial cells. Mda5 has previously been shown to specifically recognize positive-sense single-stranded RNA viruses like picornaviruses and alphaviruses [13]. Moreover, expression of TLR3 in 293 cells did not result in induction of IRF3 and NF-κB activity following HPIV3 infection (data not shown). Our observation is similar to previous studies showing the non-involvement of TLR3 during induction of innate response following infection with Sendai virus (a mouse parainfluenza virus) [20]. TLR7 (which is capable of recognizing ssRNA) also do not function as HPIV3 specific PRR in lung cells since, a) A549 cells (the lung epithelial cells utilized in our current studies) lack expression of TLR7 [21-23], b) TLR7 activating compound (e.g. R848) failed to activate TLR7 in A549 cells [22,23], and c) TLR7 is not required for IRF3 and NF-κB activation in Sendai virus (a mouse parainfluenza virus) infected A549 cells [21]. Our future studies will be directed in identifying and characterizing additional PRRs that may play a role in activating innate response following HPIV3 infection.

## Abbreviations

HPIV3: human parainfluenza virus type 3; RIGI: retinoic acid inducible gene-I; TNF: tumor necrosis factor-α; IFN: interferon; ISG15: interferon stimulated gene-15; IRF3: interferon regulatory factor 3.

## Competing interests

The authors declare that they have no competing interests.

## Authors' contributions

AS and SB designed the experiments and prepared the manuscript. AS performed the experiments. All authors read and approved the final manuscript.
